# Cytogenetic Evidence Clarifies the Phylogeny of the Family Rhynchocyclidae (Aves: Passeriformes)

**DOI:** 10.3390/cells10102650

**Published:** 2021-10-04

**Authors:** Rafael Kretschmer, Ismael Franz, Marcelo Santos de Souza, Analía Del Valle Garnero, Ricardo José Gunski, Edivaldo Herculano Corrêa de Oliveira, Rebecca E. O’Connor, Darren K. Griffin, Thales Renato Ochotorena de Freitas

**Affiliations:** 1 School of Biosciences, University of Kent, Canterbury CT2 7NJ, UK; rafa.kretschmer@hotmail.com (R.K.); rebeckyoc@gmail.com (R.E.O.); 2 Laboratório de Citogenética e Evolução, Departamento de Genética, Instituto de Biociências, Universidade Federal do Rio Grande do Sul, Porto Alegre 91509-900, RS, Brazil; thales.freitas@ufrgs.br; 3 Departamento de Zoologia, Instituto de Biociências, Universidade Federal do Rio Grande do Sul, Porto Alegre 91509-900, RS, Brazil; ismaelfranz@gmail.com; 4 Laboratório de Diversidade Genética Animal, Universidade Federal do Pampa, São Gabriel 97300-162, RS, Brazil; marcelodesouzabio@gmail.com (M.S.d.S.); analiagarnero@unipampa.edu.br (A.D.V.G.); ricardogunski@unipampa.edu.br (R.J.G.); 5 Instituto de Ciências Exatas e Naturais, Universidade Federal do Pará, Belém 66075-110, PA, Brazil; ehco@ufpa.br; 6 Laboratório de Cultura de Tecidos e Citogenética, SAMAM, Instituto Evandro Chagas, Ananindeua 67030-000, PA, Brazil

**Keywords:** phylogenetic relationships, chromosomal rearrangements, cytotaxonomy, passerines, tyrant flycatchers

## Abstract

The phylogenetic position and taxonomic status of Rhynchocyclidae (Aves: Passeriformes) have been the subject of debate since their first description. In most models, Rhynchocyclidae represents a subfamily-level taxon placed within the Tyrant Flycatchers (Tyrannidae). Considering that this classification does not include cytotaxonomic characters, we tested the hypothesis that the chromosome organization of Rhynchocyclidae members differs from that of Tyrannidae. Hence, we selected two species, *Tolmomyias sulphurescens*, and *Pitangus sulphuratus*, representing Rhynchocyclidae and Tyrannidae, respectively. Results revealed a diploid number (2n) of 60 in *T*. *sulphurescens* and 2n = 80 in *P*. *sulphuratus*, indicating significant chromosomal differences. Chromosome mapping of *Gallus gallus* (GGA) and *Taeniopygia guttata* bacterial artificial chromosome (BAC) corresponding to chromosomes GGA1-28 (except 16) revealed that the genome evolution of *T*. *sulphurescens* involved extensive chromosome fusions of macrochromosomes and microchromosomes. On the other hand, *P*. *sulphuratus* retained the ancestral pattern of organization of macrochromosomes (except the centric fission involving GGA1) and microchromosomes. In conclusion, comparing our results with previous studies in Tyrant Flycatchers and allies indicates that *P*. *sulphuratus* has similar karyotypes to other Tyrannidae members. However, *T*. *sulphurescens* does not resemble the Tyrannidae family, reinforcing family status to the clade named Rhynchocyclidae.

## 1. Introduction

The phylogenetic position and taxonomic status of the flycatcher lineage named Rhynchocyclidae (Aves: Passeriformes) have been debated since their proposition. In most classifications, it represents a subfamily placed within the Tyrant Flycatchers (Tyrannidae), composing the most diverse Neotropical family of suboscine passerines [1]. Tyrannidae “lato sensu” exhibits high degrees of morphological, ecological, and behavioral diversity, drawing the attention of several phylogenetic studies [1,2,3,4,5]. However, some aspects of their relationships and classification remain controversial. In a recent study of a massive dataset resulting in a complete super-tree of the *Tyranni passerines* (“suboscines”), Tyrannidae and Rhynchocyclidae were recovered as monophyletic and well-supported sister clades which diverged 21.8 million years ago (Mya) [6]. In addition, Onychorhynchidae, Oxyruncidae, Pipritidae, Platyrinchidae, and Tachurisidae were also well-supported as separated families [6].

Most of the Tyrant Flycatchers and allies have their diploid number (2n) close to 80. However, an interesting 2n variation has been found, ranging from 2n = 60 in *Platyrinchus mystaceus* (Platyrinchidae) to 2n = 84 in *Cnemotriccus fuscatus* (Tyrannidae: *Fluvicolinae*) (Table 1). The typical 2n of Tyrant Flycatchers and allies represents the most frequent finding in other Passeriformes and the whole Aves. According to Degrandi et al. [7], approximately 61% of avian species have a karyotype description of 2n between 76 and 82 chromosomes. Considering that the putative avian ancestral karyotype (PAK) had 2n = 80 [8], the deviation from this diploid number resulted from different chromosomal rearrangements. Usually, the decrease or increase of the diploid number can result from fusion or fission events, respectively [9]. While in some orders, the 2n was increased, such as in Piciformes (up to more than 100 chromosomes) [10], decreased, such as in Psittaciformes [11,12], in Passeriformes, the ancestral 2n is conserved in most species [7]. Unfortunately, due to the poor quality of G-banding in macrochromosomes and the small size of microchromosomes, classical cytogenetic techniques have provided limited information concerning the process of karyotypic evolution in birds.

Fortunately, the use of fluorescence in situ hybridization (FISH) can overcome these limitations. Experiments of comparative chromosome painting with several sets of probes have been performed in different bird lineages to clarify the chromosomal rearrangements involved in the reorganization of avian karyotypes [7,8,18]. Among them, the most widely used sets were from *Gallus gallus* (GGA) and *Leucopternis albicollis* [7,8,18,19].

Chromosome painting studies using macrochromosomes probes from these species have been applied only in four Tyrant Flycatcher species, *Elaenia spectabilis* (2n = 80), *Serpophaga subcristata* (2n = 82), *Pitangus sulphuratus* (2 = 80), and *Satrapa icterophrys* (2n = 82) [14,15]. These studies have revealed the fission of chicken chromosome 1 in all these species, which can be considered as a candidate synapomorphy for Passeriformes since it was found in all Passeriformes studied so far [7,18]. In addition, *S*. *icterophrys* has fission in chicken chromosome 2 [15]. Compared to PAK [8], it is likely that the karyotype evolution of Tyrant Flycatchers involved mostly fissions events. However, only the macrochromosomes have been analyzed in this lineage.

Although macrochromosomes represent approximately 77% of the average avian genome size, microchromosomes contain around 50% of the avian genes [20,21,22]. Despite the importance of microchromosomes, their organization was studied in few avian orders, and interchromosomal rearrangements involving them have been found only in few orders [12,23,24,25,26]. Among the Passeriformes, only five species have been investigated: four oscine members, *Taeniopygia guttata*, *Turdus merula*, *Serinus canaria*, and *Sicalis flaveola*, and one suboscines member, *Willisornis vidua* [24,27,28]. No evidence of interchromosomal rearrangements involving the microchromosomes was observed in these oscine species [24,28]. On the other hand, a fusion involving *G*. *gallus* chromosome pairs 5 and 17 was observed in *W*. *vidua* [28]. Apart from this, the organization of the microchromosomes in Tyrannidae and Rhynchocyclidae flycatchers remains virtually unknown, as in most birds. In the latter, even the macrochromosomes organization has not been explored. Therefore, further studies must search for chromosome signatures to understand the phylogenetic relationship and chromosome organization in this group.

This study aimed to compare the chromosome organization of members belonging to Rhynchocyclidae and Tyrannidae to verify if cytotaxonomic characters corroborate the family-level status of Rhynchocyclidae. With this in mind, we selected *Tolmomyias sulphurescens* as a representative member of Rhynchocyclidae and *P*. *sulphuratus* from Tyrannidae. *T*. *sulphurescens* was selected randomly from the Rhynchocyclidae members and the *P*. *sulphuratus* was selected because it has a typical karyotype for Tyrannidae members, as indicated on previously study [15]. Our results indicated considerable chromosomal differences between both species, and the comparison with previous studies in Tyrant Flycatchers and allies reinforces that *T*. *sulphurescens* does not resemble the family Tyrannidae.

## 2. Materials and Methods

### 2.1. Specimens and Chromosome Preparation

Two male individuals of *T*. *sulphurescens* (from Porto Vera Cruz city, Rio Grande do Sul State, Brazil) and one male of *P*. *sulphuratus* (from São Gabriel city, Rio Grande do Sul State, Brazil) were used in this study. The animals were captured in their natural environment using mist nests (permissions 026/2012 and 018/2014—CEUA/Universidade Federal do Pampa, and SISBIO 33860-3—ICMBio). From each individual, skin biopsies were used to establish fibroblast cell culture, according to Furo et al. [11]. The chromosome preparations were obtained by standard arrest with colcemid (1 h), hypotonic treatment with 0.075 M KCl (15 min), and cell fixation in methanol–acetic acid (3:1).

### 2.2. Giemsa Staining

Chromosome morphology and diploid numbers (2n) were determined based on the analysis of at least 30 stained metaphases (5% Giemsa in phosphate buffer pH 6.8 for 5 min) from each individual. Karyotypes were arranged according to chromosome size and morphology following Guerra [29].

### 2.3. Fluorescence In Situ Hybridization (FISH)

Two *G*. *gallus* or *T*. *guttata* Bacterial artificial chromosome (BAC) probes corresponding to each pair GGA1-28 (except GGA16) were selected and positioned as close as possible to the end of each chromosome arms and applied to metaphases of *T*. *sulphurescens* (Table S1). In *P*. *sulphuratus*, only BAC probes for microchromosomes GGA11-28 (except GGA16) were used because the macrochromosomes have been previously published by Rodrigues et al. [15] (Table S1). The GGA16 was not tested in both species because there are no BAC probes available for this chromosome. Most of the BAC probes were chosen from *G*. *gallus*, however, for some chromosomes, the *T*. *guttata* probes give stronger signals than *G*. *gallus* ones in Passeriformes species. In this case, we chose *T*. *guttata* probes. The preparation of probes and hybridization were performed following O’Connor et al. [24]. At least 15 metaphase spreads per individual and for each probe were analyzed to confirm the FISH results. The chromosomes were counterstained with DAPI (blue), and the BAC probes were labeled with (Texas Red) (red) or FITC (green).

Although we used BAC probes from *G*. *gallus* and *T*. *guttata*, all karyotype comparisons were performed with the chicken karyotype, since it has a similar karyotype to the ancestral avian lineage (Palaeognathae) [30] and is the reference in cytogenetics and genetics studies.

## 3. Results

### 3.1. Karyotype Description

**Figure 1:**
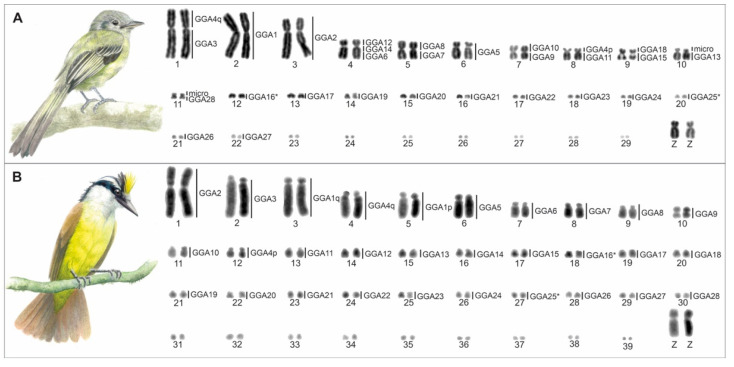
Conventionally stained complete karyotypes of *Tolmomyias sulphurescens* with 2n = 60 (**A**) and *Pitangus sulphuratus* with 2n = 80 (**B**) showing homologies to *Gallus gallus* (right). The homologies of *P*. *sulphuratus* macrochromosomes were based on Rodrigues et al. [15].

The flycatchers analyzed here showed distinct karyotypes. *T*. *sulphurescens* had a lower diploid number (2n = 60), consisting of 11 macrochromosomes, including the sex chromosomes, and 19 microchromosomes (Figure 1A). On the other hand, *P*. *sulphuratus* had a typical avian diploid number (2n = 80), consisting of 12 macrochromosomes, including the sex chromosomes, and 28 microchromosomes (Figure 1B). The Z chromosome is a submetacentric in both species.

### 3.2. Fluorescence In Situ Hybridization (FISH) Experiments

**Figure 2:**
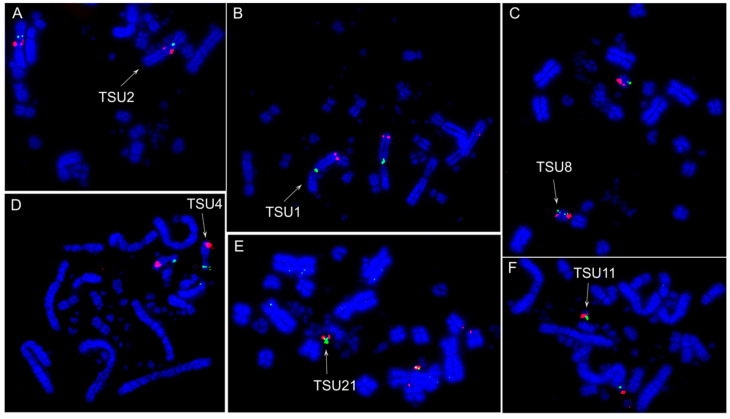
Representative cross-species hybridization results using *G*. *gallus* (CH261) and *T*. *guttata* (TGMCBA) BAC probes on *Tolmomyias sulphurescens* metaphases. (**A**) *G*. *gallus* macrochromosome 1 CH261-36B5 (green) and CH261-118M1 (red); (**B**) *G*. *gallus* macrochromosome 3 TGMCBA-295P5 (green) and CH261-169K18 (red); (**C**) *G*. *gallus* macrochromosome 4 CH261-83E1 (green) and *G*. *gallus* microchromosome 11 CH261-121N21 (red); (**D**) *G*. *gallus* microchromosome 12 CH261-60P3 (green), and *G*. *gallus* macrochromosome 6 CH261-49F3 (red); (**E**) *G*. *gallus* microchromosome 26 CH261-186M13 (green) and CH261-170L23 (red); (**F**) *G*. *gallus* microchromosome 28 CH261-64A15 (green) and CH261-72A10 (red).

**Figure 3:**
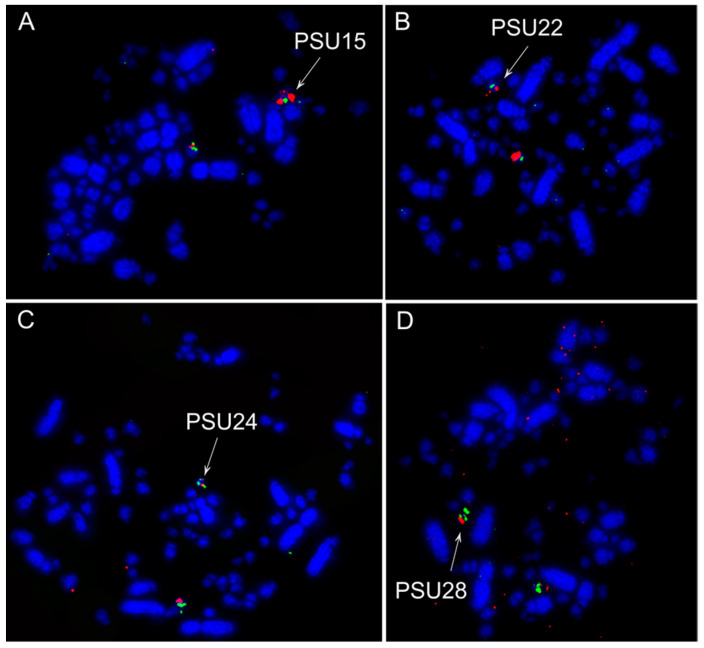
Representative cross-species hybridization results using *G*. *gallus* (CH261) and *T*. *guttata* (TGMCBA) BAC probes on *Pitangus sulphuratus* metaphases. (**A**) *G*. *gallus* microchromosome 13 CH261-115I12 (green) and TGMCBA-321B13 (red); (**B**) *G*. *gallus* microchromosome 20 TGMCBA-250E3 (green) and TGMCBA-341F20 (red); (**C**) *G*. *gallus* microchromosome 22 CH261-40J9 (green) and CH261-18G17 (red); (**D**) *G*. *gallus* microchromosome 26 CH261-186M13 (green) and CH261-170L23 (red).

The hybridizations of BAC probes from *G*. *gallus* chromosome 1–28 (except 16) revealed extensive chromosome rearrangements in *T*. *sulphurescens*. Out of 27 chromosomes tested, only 12 chromosomes (GGA1, 2, 5, 17, and 19–27) were not involved in interchromosomal rearrangements (Figure 1A and Figure 2). The following associations were observed in *T*. *sulphurescens*: GGA3/4q (TSU 1), GGA4p/11 (TSU 8), GGA6/14/12 (TSU 4), GGA7/8 (TSU 5), GGA9/10 (TSU 7), GGA15/18 (TSU 9), GGA13/micro (TSU 10); GGA28/micro (TSU 11). It was clear that *G*. *gallus* chromosomes 13 and 28 are fused with other elements, because the signals were observed in larger chromosomes, if we compare with the sizes of *G*. *gallus* chromosomes 13 and 28. Probably, one of the microchromosome pairs not used in our analysis (GGA16, 29–38) fused with the GGA13 and 28 to originate the TSU10 and 11, respectively. On the other hand, there was no evidence of rearrangements involving microchromosomes in *P*. *sulphuratus* (Figure 1B and Figure 3).

## 4. Discussion

Passerines usually show a 2n close to 80 chromosomes, however, Tyrant Flycatchers and allies have a remarkable variation, from 2n = 60 in *P*. *mystaceus* (Platyrinchidae) to 2n = 84 in *C*. *fuscatus* (Tyrannidae) [13]. To the best of our knowledge, the karyotype of *T*. *sulphurescens* (2n = 60) is described here for the first time, and the karyotype of *P*. *sulphuratus* (2n = 80) agrees with its recent description [15]. Hence, our results reinforce the chromosomal variation observed previously among Tyrant Flycatchers and allies.

**Figure 4:**
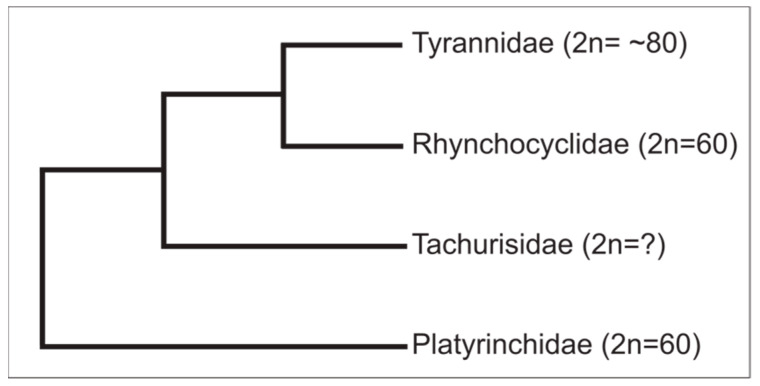
Chromosomal data plotted on a phylogenetic tree adapted from Harvey et al. [6]. The diploid numbers (2n) for Rhynchocyclidae and Platyrinchidae are based in the data obtained to *Tolmomyias sulphurescens* (present study) and *Platyrinchus mystaceus* [13]. The 2n for Tyrannidae is considered as ~80 because most of the species karyotyped so far in this family have approximately 80 chromosomes (Table 1).

Low diploid numbers, such as seen in *T*. *sulphurescens* and *P*. *mystaceus*, both with 60 chromosomes, are rare among Passeriformes [7]. This may indicate a common ancestor in these species. According to the phylogenetic relationships found by Harvey et al. [6], Rhynchocyclidae and Tyrannidae are sister groups to Tachurisidae, and Platyrinchidae is the sister group to the former families (Figure 4). Tachurisidae represents a monotypic family, with *Tachuris rubrigastra* as the unique member. Despite the fact that there are no cytogenetic studies in this species, and considering the phylogenetic relationships proposed by Harvey et al. [6], a parsimony-based view would predict it has a low diploid number, similar to *T*. *sulphurescens* and *P*. *mystaceus*. Alternatively, the diploid number found in *T*. *sulphurescens* (Rhynchocyclidae) and *P*. *mystaceus* (Platyrinchidae) may be a result of independent karyotype reorganization. However, considering that low diploid number is rare in birds, especially Passeriformes, and both species have similar chromosomal morphology, this alternative seems not to be parsimoniously supported.

Unfortunately, few species of Tyrant Flycatchers and allies have been karyotyped (Table 1). Among the Rhynchocyclidae members, the first karyotype description is from *T*. *sulphurescens* (present study). However, Gunski et al. [13] described in their paper unpublished data that *Corythopis delalandi*, another Rhynchocyclidae member, has a similar karyotype to *P*. *mystaceus*, and consequently, similar to *T*. *sulphurescens*. These observations indicate that low diploid number may be a common feature among the Rhynchocyclidae members.

Although *T*. *sulphurescens* and *P*. *sulphuratus* have a similar number of macrochromosomes, 11 and 12 pairs, respectively, they differ substantially in the number of microchromosomes, 22 pairs in *T*. *sulphurescens* and 28 in *P*. *sulphuratus*, highlighting the role of chromosome rearrangements involving microchromosomes in *T*. *sulphurescens*. In fact, our molecular cytogenetic results revealed that several microchromosomes and macrochromosomes were involved in fusion events in *T*. *sulphurescens*. At the same time, no evidence of this type of rearrangement was found in *P*. *sulphuratus*. Fusion involving microchromosomes are rare events in birds and have been found extensively in few avian orders [24,25,26]. Here, we also demonstrated extensively fusion involving microchromosomes in Passeriformes, e.g., *T*. *sulphurescens*. Recent studies demonstrated that four songbirds, *Taeniopygia guttata*, *Turdus merula*, *Serinus canaria*, and *Sicalis flaveola* [24,28], and here *P*. *sulphuratus*, have the ancestral pattern of microchromosome organization. Hence, interchromosomal rearrangements involving microchromosomes represent an unusual feature of *T*. *sulphurescens* and probably also in closely related species, e.g., Rhynchocyclidae, Tachurisidae, and Platyrinchidae (Figure 4).

Considering the high chromosomal differences between *T*. *sulphurescens* and *P*. *sulphuratus*, our data indicate that, at least from the cytogenetic point of view, these species belong to different lineages. Hence, our findings reinforce the diagnosability and recognition of the Rhynchocyclidae family [2,4,5,6,31,32,33,34].

A recent study found evidence that the microchromosomes GGA10, GGA13, and GGA14 are more prone to interchromosomal rearrangements than others. Moreover, only GGA10 was supported with statistical significance after adjusting the number of tests performed [25]. Here, we reinforce these findings since, in *T*. *sulphurescens*, these microchromosomes were also involved in fusion events (Figure 1A). In addition, we reinforce that the microchromosomes GGA22, GGA24, GGA26, and GGA27 seem not prone to interchromosomal rearrangements.

The most unexpected finding in *T*. *sulphurescens* was that the *G*. *gallus* chromosome 1 (GGA1) probe hybridizes in only one pair: up to now, all Passeriformes analyzed showed centric fission in this chromosome [18]. Hence, two hypotheses may be highlighted: (1) the GGA1 as an entire chromosome represents a plesiomorphic (ancestral) character retained in *T*. *sulphurescens*, or (2) *T*. *sulphurescens* had the fusion of GGA1p and GGA1q, restoring the ancestral character. Considering that the centric fission in this chromosome was found in all Passeriformes and Psittaciformes (Passeriformes sister group) species previously studied [7,18], it is likely that the second hypothesis is more plausible.

Together with previous studies, our results indicate that the karyotype of Tyrannidae evolved with few interchromosomal rearrangements. On the other hand, these rearrangements are likely to be the most frequent events in Rhynchocyclidae when compared to PAK [8]. In general, chromosomal rearrangements occur in breakpoint regions, usually associated with genomic features, including transposable elements, and conserved noncoding elements [35,36]. Hence, the presence of these genomic features in Rhynchocyclidae members and not in Tyrannidae members might facilitate the extensive chromosome reorganization in the former.

The Passeriformes order represents more than half of all living birds and displays great diversity in richness within subgroups, morphological and ecological diversification [37,38]. Interestingly, the rise of this great diversity was not accompanied by a high rate of interchromosomal rearrangements (e.g., fissions and fusions) [7,18], except in *T*. *sulphurescens* (present study). This may indicate that, in general, the maintenance of the ancestral pattern of karyotype in Passeriformes was crucial to the successful diversification seen in this clade. However, it remains unclear why extensive chromosome rearrangements evolved in some avian lineages, such as in *T*. *sulphurescens* and probably in other closely related species, while other Passeriformes retained the ancestral pattern of karyotype organization (~80 chromosomes). Moreover, it is known that Passeriformes underwent a high number of intrachromosomal rearrangements, such as paracentric and pericentric inversions [39,40].

In conclusion, our results indicate that the chromosome evolution of *T*. *sulphurescens* involved extensive chromosome fusions of macrochromosomes and microchromosomes, while *P*. *sulphuratus* retained the ancestral pattern of organization of macrochromosomes and microchromosomes, except for the fission of *G*. *gallus* chromosome 1. The comparison of our results with previous studies in Tyrant Flycatchers and allies indicates that the karyotype of *P*. *sulphuratus* is similar to other Tyrannidae members, however, *T*. *sulphurescens* does not resemble the Tyrannidae family, reinforcing the status of the family to Rhynchocyclidae. The high chromosomal differences observed in Tyrant Flycatchers and allies make these birds an ideal model to investigate the role of chromosomal rearrangements in speciation and to detect what contributed to chromosomal rearrangements Rhynchocyclidae, but not in Tyrannidae members.

## Figures and Tables

**Table 1:** Available cytogenetic data for species of Tyrant Flycatchers and related families (classification according to the relationships found by Harvey et al. [6]).

Species	2n	Family	Reference
*Platyrinchus mystaceus*	2n = 60	Platyrinchidae	[13]
*Elaenia parvirostris*	2n = 78	Tyrannidae	[13]
*Elaenia spectabilis*	2n = 80	Tyrannidae	[14]
*Serpophaga subcristata*	2n = 82	Tyrannidae	[15]
*Pitangus sulphuratus*	2n = 80	Tyrannidae	[15]
*Tyrannus melancholicus*	2n = 78	Tyrannidae	[13]
*Tyrannus savana*	2n = 78	Tyrannidae	[13]
*Myiarchus ferox*	2n = 76	Tyrannidae	[13]
*Knipolegus cyanirostris*	2n = 78	Tyrannidae	[16]
*Satrapa icterophrys*	2n = 82	Tyrannidae	[15]
*Cnemotriccus fuscatus*	2n = 84	Tyrannidae	[13]
*Empidonax alnorum*	2n = 82	Tyrannidae	[17]
*Empidonax flaviventris*	2n = 82	Tyrannidae	[17]
*Empidonax hammondii*	2n = 82	Tyrannidae	[17]
*Empidonax minimus*	2n = 82	Tyrannidae	[17]
*Empidonax traillii*	2n = 82	Tyrannidae	[17]

## Data Availability

All the data supporting our findings are contained within the manuscript.

## Supplementary Materials

The following are available online at https://www.mdpi.com/article/10.3390/cells10102650/s1, Table S1: List of BAC applied to *Tolmomyias sulphurescens* (TSU) and *Pitangus sulphuratus* (PSU).
